# Information-Theoretic Reliability Analysis of Linear Consecutive r-out-of-n:F Systems and Uniformity Testing

**DOI:** 10.3390/e27060590

**Published:** 2025-05-31

**Authors:** Ghadah Alomani, Faten Alrewely, Mohamed Kayid

**Affiliations:** 1 Department of Mathematical Sciences, College of Science, Princess Nourah bint Abdulrahman University, P.O. Box 84428, Riyadh 11671, Saudi Arabia; 2 Department of Mathematics, College of Science, Jouf University, P.O. Box 2014, Sakaka 72388, Saudi Arabia; 3 Department of Statistics and Operations Research, College of Science, King Saud University, P.O. Box 2455, Riyadh 11451, Saudi Arabia; drkayid@ksu.edu.sa

**Keywords:** consecutive r-out-of-n:F systems, extropy, goodness-of-fit test, Shannon entropy, stochastic orders

## Abstract

This paper explores the reliability of linear consecutive r-out-of-n:F systems from an information-theoretic perspective, with a particular focus on testing for uniformity. At the heart of the study is extropy, a complementary measure to entropy that we use to gain deeper insights into the uncertainty associated with system lifetimes. We begin by deriving general expressions for extropy in these systems and examine how it behaves under different component lifetime distributions, particularly highlighting the role of heterogeneity. Theoretical bounds are developed, along with new characterization results, shedding light on the unique properties of the uniform distribution within this framework. To bridge theory and application, we propose a nonparametric estimator for extropy and build a new test statistic to assess uniformity. The effectiveness of this test is evaluated through comprehensive simulation studies, where we compare its power against several well-known alternatives across a range of scenarios. Overall, our findings offer both theoretical contributions to the understanding of information measures in reliability analysis and practical tools for statistical testing in applied settings.

## 1. Introduction

The quantification of uncertainty in probability distributions is a fundamental aspect of information theory, which provides essential tools for measuring and analyzing randomness. Among its key metrics, the concept of Shannon differential entropy holds particular significance. In his seminal work, Shannon [1] introduced entropy as a measure of uncertainty. For a non-negative random variable X with a probability density function (pdf) h(x), the Shannon differential entropy is defined as H(X)=E(−log⁡h(X)), where E(⋅) denotes the expectation operator and $\log\left(\cdot \right)$ represents the natural logarithm, provided the expectation is well-defined. Recently, Lad et al. [2] introduced extropy, a novel measure of uncertainty that serves as a dual counterpart to entropy. For a non-negative random variable X with pdf h(x) and cumulative distribution function (cdf) H(x)=(X≤x), the extropy is defined as follows:

(1) $$J\left(X\right)=-\frac{1}{2}\int_{0}^{\infty }h^{2}\left(x\right)dx=-\frac{1}{2}E\left[h\left(H^{-1}\left(U\right)\right)\right],$$

where U is a uniform random variable on the interval [0,1], $H^{-1}\left(u\right)=inf\left\{x:H\left(x\right)\geq u\right\},u\in \left[0,1\right],$ $u\in \left[0,1\right],$ signifying the quantile function of H.

These measures have proven highly effective in quantifying uncertainty, making them valuable across various disciplines. One significant statistical application of extropy involves evaluating forecasting distributions using the total log scoring rule. For a deeper exploration of its theoretical and applied aspects, notable references include Agro et al. [3], Capotorti et al. [4], and Gneiting and Raftery [5].

For an absolutely continuous non-negative random variable X with cdf $H\left(x\right)$ and reversed hazard rate function $\tau \left(x\right)=h\left(x\right)/H\left(x\right)$, an alternative representation of extropy can be expressed as follows:

(2) $$J\left(X\right)=-\frac{1}{4}E_{22}\left[\tau \left(X\right)\right],$$

where $E_{22}$ denotes the expectation with respect to the pdf (Toomaj et al. [6]):

(3) $$h_{22}\left(x\right)=2h\left(x\right)H\left(x\right),x>0.$$

The density function in (3) represents the probability density of the maximum of two independent and identically distributed (i.i.d.) random variables. Several properties and statistical applications of the extropy measure in (1) have been extensively explored by Lad et al. [2] and Yang et al. [7].

Research on consecutive r-out-of-n systems has gained significant attention in reliability analysis, owing to their broad applicability in engineering and infrastructure systems. These systems are typically classified as either failure (*F*) or good (*G*) structures, depending on their operational principles. Between these, linear consecutive r-out-of-n:F systems have been extensively studied due to their practical relevance. These systems consist of n identical, independent components arranged in a linear sequence, and they fail if and only if at least r consecutive components fail. Conversely, a linear consecutive r-out-of-n:G system remains functional only if at least r consecutive components remain operational.

For the remainder of this discussion, we focus on the linear consecutive r-out-of-n:*F* system, given its widespread implementation in telecommunication networks, street parking management, microwave relay stations, vacuum systems, and oil pipeline infrastructure. A representative example is an *n*-station oil pipeline system, where each station is spaced 100 km apart and has the capacity to pump oil over 400 km. This system exhibits a 4-out-of-*n*:*F* reliability configuration, implying that the failure of any four consecutive stations leads to complete system failure. Notably, special cases of consecutive r-out-of-n:F systems correspond to well-known reliability structures: the 1-out-of-*n*:*F* system is equivalent to a series system, whereas the n-out-of-n:F system corresponds to a parallel system. The reliability characteristics of these systems have been rigorously analyzed under various modeling assumptions (see, e.g., Jung and Kim [8], Shen and Zuo [9], Kuo and Zuo [10], Chang et al. [11], Boland and Samaniego [12], and Eryılmaz [13,14]).

This paper examines the extropy properties of consecutive r-out-of-n:F systems, assuming that component lifetimes are independent and identically distributed (i.i.d.). The study of information measures related to ordered data has been a key area of research in reliability and information theory, as reflected in extensive studies, such as those in references [15,16,17,18,19].

In recent years, extropy has gained prominence as a measure of uncertainty, with several researchers contributing to its development. Notable studies include those by Lad et al. [2], Qiu [20], and Qiu and Jia [21,22]. Qiu et al. [20] conducted a comprehensive analysis of extropy in the context of order statistics and record values, shedding light on its uniqueness and monotonic properties. These aspects were further explored in later studies by Qiu and Jia [22]. Additional contributions in this area have examined extropy within different statistical and reliability frameworks. Research by Kayid and Alshehri [23] investigated its properties in relation to system lifetimes, while Shrahili and Kayid [24] explored the residual extropy of order statistics. Related studies by Alshehri et al. [25] expanded on these findings, offering deeper insights into its applications. Further work has also examined the extropy of past lifetimes in coherent systems, particularly in scenarios where components remain inactive for a specified period [26]. More recently, Alrewely and Kayid [27] explored the extropy of consecutive r-out-of-n:G systems, providing a framework for theoretical and practical applications. They derived expressions for system lifetime extropy and evaluated it across various distributions. They also proposed a novel test statistic for exponentiality, with performance assessments highlighting its effectiveness in specific contexts. Building on these foundational studies, this paper aims to extend the understanding of extropy, focusing on its behavior within linear consecutive r-out-of-n:F systems.

The remainder of this paper is organized as follows. Section 2 derives the extropy of a consecutive r-out-of-n:F system with lifetime $T_{k|n:F}$ for an arbitrary lifetime distribution. This is achieved by establishing a relationship with the extropy of a comparable system in which component lifetimes follow a uniform distribution. This connection provides a broader perspective on extropy measures across different distributional settings. Section 3 addresses the challenge of obtaining explicit expressions for the extropy of order statistics, which is often difficult in various statistical models. To mitigate this, we derive bounds for the extropy of consecutive r-out-of-n systems. Section 4 presents a characterization of extropy in these systems, with particular emphasis on the uniform distribution. Section 5 includes computational results to validate the theoretical findings. Specifically, we introduce a nonparametric estimator for the extropy of consecutive systems and apply it to a hypothesis test for the standard uniform distribution. Finally, Section 6 summarizes the key findings and outlines potential directions for future research.

## 2. Extropy of Consecutive r-out-of-n:F System

In this section, we examine the extropy properties of consecutive r-out-of-n:F systems, where the system fails as soon as at least r consecutive components fail. To analyze this, let $X_{1},X_{2},\cdots ,X_{n}$ denote the lifetimes of the system’s components, each following a common cumulative distribution function (cdf) H(x) and probability density function (pdf) h(x). The overall system lifetime is denoted by $T_{r|n:F}$. When 2r≥n, Navarro and Eryılmaz [28] established that the system’s reliability function can be expressed as follows:

$$H_{r|n:F}(t)=(n-r+1)H^{r}(t)-(n-r)H^{r+1}(t),t>0.$$

This formulation provides a fundamental relationship for evaluating the reliability of consecutive r-out-of-n:*F* systems under this condition. It follows that

(4) $$\begin{array}{ll} h_{r|n:F}(t) & =r(n-r+1)H^{r-1}(t)h(t)-\left(r+1\right)\left(n-r\right)H^{r}\left(t\right)h\left(t\right)\\ & =(n-r+1)h_{r:r}(t)-(n-r)h_{r+1:r+1}(t),t>0, \end{array}$$

where $h_{j:j}(t)=jh(t)H^{j-1}(t)$ is the pdf of the parallel system with lifetime $X_{j:j}=max\left(X_{1},\cdots ,X_{j}\right)$. We will use the probability integral transform $V_{r|n:F}=H\left(T_{r|n:F}\right)$ to find a formula for the extropy of a consecutive r-out-of-n:F system with lifetime $T_{r|n:F}$. This transform changes the original lifetimes $X_{i}$ into new lifetimes $V_{i}=H\left(X_{i}\right)$ that are uniformly distributed in [0,1]. When 2r is greater than or equal to n, the pdf $U_{r|n:F}$ is

(5) $$g_{r|n:F}\left(v\right)=r\left(n-r+1\right)v^{r-1}-\left(r+1\right)\left(n-r\right)v^{r},\;for\;all\;0<v<1.$$

The following theorem provides an explicit expression for the extropy of a consecutive r-out-of-n:F system with lifetime $T_{r|n:F}$. Since the proof of this result follows directly from established principles, it is omitted for brevity.

**Theorem** **1.** *For 2r≥n, the extropy of $T_{r|n:F}$ can be expressed as follows:*

(6) $$J\left(T_{r|n:F}\right)=-\frac{1}{2}\int_{0}^{1}g_{r|n:F}^{2}\left(v\right)h\left(H^{-1}\left(v\right)\right)dv,$$

*where* $g_{r|n:F}(v)$ *is given by (5).*

**Proof.**  By applying the transformation v=F(x), and referencing Equations (1) and (4), we can derive the following expressions:

$$\begin{array}{ll} J\left(T_{r|n:F}\right) & =-\frac{1}{2}\int_{0}^{\infty }h_{r|n:F}^{2}\left(t\right)dt\\ & =-\frac{1}{2}\int_{0}^{\infty }{h^{2}\left(x\right)\left(r\left(n-r+1\right)H^{r-1}\left(t\right)-\left(r+1\right)\left(n-r\right)H^{r}\left(t\right)\right)}^{2}dt\\ & =-\frac{1}{2}\int_{0}^{1}{h\left(H^{-1}\left(v\right)\right)\left(r\left(n-r+1\right)v^{r-1}-\left(r+1\right)\left(n-r\right)u^{r}\right)}^{2}dv\\ & =-\frac{1}{2}\int_{0}^{1}g_{r|n:F}^{2}\left(v\right)h\left(H^{-1}\left(v\right)\right)dv, \end{array}$$

which completes the proof. □

An alternative representation derived from Equation (4) is given below:

(7) $$\begin{array}{ll} J\left(T_{r|n:F}\right) & =-\frac{1}{2}\int_{0}^{\infty }h_{r|n:F}^{2}(t)dt\\ & =-\frac{1}{2}\int_{0}^{1}{\left(\left(n-r+1)h_{r:r}(t)-(n-r)h_{r+1:r+1}(t\right)\right)}^{2}dt\\ & =(n-r+1{)}^{2}J\left(X_{r:r}\right)+(n-r{)}^{2}J\left(X_{r+1:r+1}\right)-2(n-r+1)(n-r)J\left(X_{r:r}X_{r+1:r+1}\right), \end{array}$$

where

$$J\left(X_{r:r}X_{r+1:r+1}\right)=-\frac{1}{2}\int_{0}^{\infty }h_{r:r}\left(t\right)h_{r+1:r+1}\left(t\right)dt,$$

represents the inaccuracy measure of $h_{r:r}(x)$ with respect to $h_{r+1:r+1}(x)$, or vice versa.

As an application of representation (6), we now present the following example.

**Example** **1.** *Consider a linear consecutive r-out-of-n:F system with component lifetimes $X_{1},X_{2},\ldots ,X_{n}$. The system fails if and only if at least r consecutive components fail. The system’s lifetime is given by*

$$T_{r|n:F}=min\left(T^{\left[1:r\right]},T^{\left[2:r+1\right]},\ldots ,T^{\left[n-r+1:n\right]}\right)$$

*where* $T^{\left[j:m\right]}=max\left(T_{j},\ldots ,T_{m}\right)$ *for* 1≤j<m≤n. *Assuming that the lifetimes of the components follow a Weibull distribution characterized by a shape parameter α and a scale parameter of one, the cdf is given by*

$$H\left(x\right)=1-e^{-x^{\alpha }},x>0,for\;\alpha >0.$$

*It can be shown that*

$$h\left(H^{-1}(v)\right)=\alpha (1-v)(-log(1-v){)}^{\frac{\alpha -1}{\alpha }},0<v<1$$

*Recalling Equation (6), for all* 2r≥n, *the extropy of the system’s lifetime can be expressed as follows:*

$$J\left(T_{r|n:F}\right)=-\frac{\alpha }{2}\int_{0}^{1}g_{r|n:F}^{2}(v)(1-v)(-log(1-v){)}^{\frac{\alpha -1}{\alpha }}dv.$$

*Since obtaining an explicit expression for this integral is challenging, numerical methods were used to analyze the relationship between* $J\left(T_{r|n:F}\right)$ *and the shape parameter* α. *This study focused on the consecutive* r-out-of-8:F *system, with r ranging from 4 to 8*.

**Figure 1:**
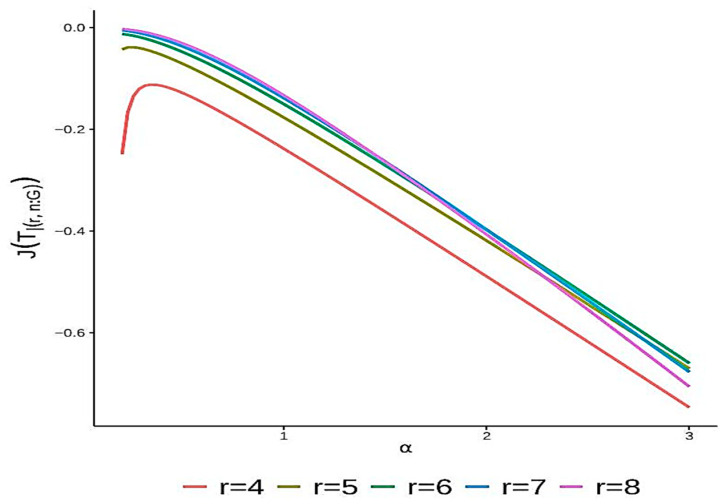
The precise values of $J\left(T_{r|n:F}\right)$ as a function of α for various values of r and n=8, as demonstrated in Example 1.

Figure 1 illustrates that the system’s extropy initially increases with increasing α before eventually decreasing again. This clearly demonstrates the influence of the shape parameter on the system’s extropy. Interestingly, no direct correlation was observed between the monotonic behavior of extropy and the number of functioning components.

Consider two random variables, X and Y with pdfs $h_{X}(x)$ and $h_{Y}(x)$, and survival functions $S_{X}(x)$ and $S_{Y}(x)$, respectively. Recall that $X\leq_{\mathrm{disp}\;}Y$, i.e., X is smaller than Y in the dispersive order if and only if

(8) $$h_{X}\left(H_{X}^{-1}(v)\right)\geq h_{Y}\left(H_{Y}^{-1}\left(v\right)\right),\;\;for\;all\;0<v<1.$$

Additionally, $X\leq_{hr}Y$, in the sense that X is smaller than Y in the hazard rate order if the ratio of survival functions $S_{Y}(x)/S_{X}(x)$ is increasing in x>0. Moreover, X exhibits the decreasing failure rate (DFR) property if the ratio of the pdf to the reliability function, $h_{X}(x)/S_{X}(x)$, is decreasing in x>0. For a deeper understanding and applications of these concepts, refer to [29]. Using (6), the following theorem becomes apparent.

**Theorem** **2.** *Consider two consecutive r-out-of-n:F systems with lifetimes $T_{r|n:F}^{X}$ and $T_{r|n:F}^{Y}$ composed of n i.i.d. components with cdfs $H_{X}$ and $H_{Y}$, and pdfs $h_{X}$ and $h_{Y}$, respectively. Then, $J\left(T_{r|n:F}^{X}\right)\leq J\left(T_{r|n:F}^{Y}\right)$ for all 2r≥n, provided that $X\leq_{\mathrm{disp}\;}Y$*.

**Proof.**  By using Equation (6), we have

$$\begin{array}{ll} J\left(T_{r|n:F}^{X}\right) & =-\frac{1}{2}\int_{0}^{1}g_{r|n:F}^{2}(v)h_{X}\left(H_{X}^{-1}(v)\right)dv\\ & \leq -\frac{1}{2}\int_{0}^{1}g_{r|n:F}^{2}(v)h_{Y}\left(H_{Y}^{-1}(v)\right)dv\\ & =J\left(T_{r|n:F}^{Y}\right), \end{array}$$

where the inequality is obtained from (8), which completes the proof. □

As shown by Bagai and Kochar [30], if $X\leq_{hr}Y$ and either X or Y is DFR, then $X\leq_{disp}Y$. Considering this result and Theorem 2, the following corollary is straightforward to prove.

**Corollary** **1.** *Under the assumptions of Theorem 2, if $X\leq_{hr}Y$ and either X or Y is DFR, then $J\left(T_{r|n:F}^{X}\right)\leq J\left(T_{r|n:F}^{Y}\right)$ for all 2r≥n*.

*A notable application of (6) is comparing the extropy of consecutive r-out-of-n:F systems with independent but different component lifetime distributions, as stated in the following theorem.*

**Theorem** **3.** *Under the conditions of Theorem 2, if J(X)≤J(Y) and $\underset{A_{1}}{inf}g_{r|n:F}(v)\geq$ $\underset{A_{2}}{sup}g_{r|n:F}(v)$, for $A_{1}=\left\{v\in [0,1]:h_{X}\left(H_{X}^{-1}(v)\right)>h_{Y}\left(H_{Y}^{-1}(v)\right)\right\},$*

$$A_{2}=\left\{v\in [0,1]:h_{X}\left(H_{X}^{-1}(v)\right)\leq h_{Y}\left(H_{Y}^{-1}(v)\right)\right\},\;then\;J\left(T_{r|n:F}^{X}\right)\leq J\left(T_{r|n:F}^{Y}\right)\;for\;all\;2r\geq n.$$

**Proof.**  Since J(X)≤J(Y), from (1), we have

(9) $$J(Y)-J(X)=-\frac{1}{2}\int_{0}^{1}\zeta (v)dv\geq 0,$$

where $\zeta (v)=h_{Y}\left(H_{Y}^{-1}(v)\right)-h_{X}\left(H_{X}^{-1}(v)\right)$, for 0<v<1. From (6), we have

$$\begin{array}{ll} J\left(T_{r|n:F}^{Y}\right)-J\left(T_{r|n:F}^{X}\right) & =-\frac{1}{2}\left[\int_{0}^{1}g_{r|n:F}^{2}(v)\zeta (v)dv\right]\\ & =-\frac{1}{2}\left[\int_{A_{1}}g_{r|n:F}^{2}(v)\zeta (v)dv+\int_{A_{2}}g_{r|n:F}^{2}(v)\zeta (v)dv\right]\left(\mathrm{since}\;A_{1}\cup A_{2}=[0,1]\right)\\ & \;\geq -\frac{1}{2}\left[{\left(\underset{v\in A_{1}}{inf}g_{r|n:F}(v)\right)}^{2}\int_{A_{1}}\zeta (v)dv+{\left(\underset{v\in A_{2}}{sup}g_{r|n:F}(v)\right)}^{2}\int_{A_{2}}\zeta (v)dv\right]\\ & \left.\;\;(\mathrm{since}\;\zeta \left(v\right)<0\mathrm{in}\;A_{1}\;\mathrm{and}\;\zeta \left(v\right)\geq 0\mathrm{in}\;A_{2}\right)\\ & \;\geq -\frac{{\left(\underset{v\in A_{2}}{sup}g_{r|n:F}(v)\right)}^{2}}{2}\int_{0}^{1}\zeta (v)dv\;(\mathrm{by}\;\mathrm{the}\;\mathrm{assumption})\;\\ & \;\geq 0\;(\mathrm{by}\;(9)). \end{array}$$

So, we have $J\left(T_{r|n:F}^{X}\right)\leq J\left(T_{r|n:F}^{Y}\right)$ for 2r≥n, completing the proof. □

The subsequent example serves to illustrate the preceding theorem.

**Example** **2.** *Assume coherent systems with lifetimes $T_{2|3:F}^{X}=min\left(max\left(X_{1},X_{2}\right),max\left(X_{2},X_{3}\right)\right)$ and $T_{2|3:F}^{Y}=min\left(max\left(Y_{1},Y_{2}\right),max\left(Y_{2},Y_{3}\right)\right)$, where $X_{1},X_{2},X_{3}$ are i.i.d. component lifetimes with a common cdf $H_{X}(t)=1-e^{-2t},t>0$, and $Y_{1},Y_{2},Y_{3}$ are i.i.d. component lifetimes with the common cdf $H_{Y}(t)=1-e^{-6t},t>0$. We can easily confirm that J(X)=−0.125 and J(Y)=−0.0416, so J(X)≤J(Y). Additionally, since $A_{1}=[0,1)$ and $A_{2}=\{1\}$, we have $\underset{A_{1}}{inf}g_{V}(v)=\underset{A_{2}}{sup}g_{V}(v)=0$. Thus, Theorem 3 implies that $J\left(T_{2|3:F}^{X}\right)\leq J\left(T_{2|3:F}^{Y}\right)$*.

## 3. Bounds of Extropy of Consecutive Systems

In many cases, deriving closed-form expressions for the extropy of consecutive systems is computationally infeasible, especially when the system consists of a large number of components or when the component lifetime distributions exhibit considerable complexity. In such scenarios, bounding techniques provide a practical and effective alternative. Motivated by this challenge, we investigated the use of bounds to characterize the extropy of consecutive r-out-of-n:F systems. To this end, we established a theorem that provides bounds for the extropy of such systems. Since the proof follows directly from established principles, it is omitted for brevity.

**Theorem** **4.** *Let us consider a consecutive r-out-of-n:F system with lifetime $T_{r|n:F}$ having n i.i.d. component lifetimes with pdf h.*

(i) *If* M=h(m)<∞*, where* m=sup{x:h(x)≤M} *designates the mode of the pdf* h*, for* 2r≥n*, we have* $J\left(T_{r|n:F}\right)\geq MJ\left(U_{r|n:F}\right)$.
(ii) *For* 2r≥n, we have
  $$B^{2}J\left(X\right)\geq J\left(T_{r|n:F}\right)\geq D^{2}J\left(X\right),$$

*where* $B=\underset{v\in (0,1)}{inf}g_{r|n:F}(v)$ *and* $D=\underset{v\in (0,1)}{sup}g_{r|n:F}(v)$.

**Proof**  The proof of Part (i) is straightforward; therefore, we focused on proving Part (ii). Since $g_{r|n:F}\left(v\right)\geq \underset{v\in (0,1)}{inf}g_{r|n:F}\left(v\right)$, by recalling Equation (9), the upper bound is given by

$$\begin{array}{ll} J\left(T_{r:n|F}\right) & =-\frac{1}{2}\int_{0}^{1}g_{r|n:F}^{2}(v)h\left(H^{-1}(v)\right)dv\\ & \;\leq -\frac{{\left(\underset{v\in \left(0,1\right)}{inf}g_{r|n:F}\left(v\right)\right)}^{2}}{2}\int_{0}^{1}h\left(H^{-1}(v)\right)dv\\ & =B^{2}J\left(X\right). \end{array}$$

The lower bound can be derived in a similar manner. □

As established in Part (i) of Theorem 4, we derived a lower bound for the extropy of $T_{r|n:F}$. This bound was obtained by considering the extropy of consecutive r-out-of-n:F systems with a uniform distribution, along with the mode M of the original distribution. In Part (ii) of Theorem 4, the extropy $J\left(T_{r|n:F}\right)$ is shown to be bounded between the extropy of the individual components under certain sufficient conditions. To illustrate these lower bounds, we examined consecutive r-out-of-n:F systems consisting of mixtures of two Pareto-distributed components.

**Example** **3.** *Consider a linear consecutive 3-out-of-5:F system with lifetime*

$$T_{3|5:F}=min\left(max\left(X_{1},X_{2},X_{3}\right),max\left(X_{2},X_{3},X_{4}\right),max\left(X_{3},X_{4},X_{5}\right)\right).$$

*We assume that the component lifetimes are i.i.d. following a mixture of two Pareto distributions with parameters 2 and 1. The pdf of this mixture distribution is given by*

$$h_{m}(x)=2b(1+x{)}^{-3}+(1-b)(1+x{)}^{-2},x\geq 0,\;for\;0<b<1.$$

*It is easy to see that the mode of this distribution is 1, and therefore,* M=b+1. *Furthermore, we can calculate* $B=\underset{v\in (0,1)}{inf}g_{3|5:F}(v)=0$ *and* $D=\underset{v\in (0,1)}{sup}g_{3|5:F}(v)=1.6875$.

*Furthermore, it is apparent that* $J\left(U_{3|5:F}\right)=-0.6714$*. Moreover, one can write* $J(X)=-\left(2b^{2}+\right.$ 5b+5)/30*. Consequently, we can establish lower bounds for* $J\left(T_{3|5:F}\right)$ *based on Theorems 4. Specifically, the lower bounds based on Parts (i) and (ii) are given by*

$$J\left(T_{3|5:F}\right)\geq -0.6714(b+1)\;\;and\;\;\left(T_{3|5:F}\right)\geq -0.095\left(2b^{2}+5b+5\right),$$

**Figure 2:**
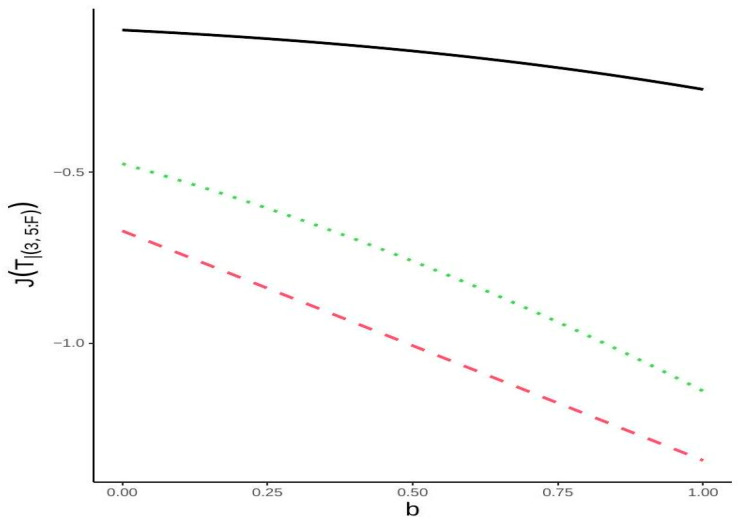
The exact values of $J\left(T_{r|n:F}\right)$ (black color) and lower bounds from Theorem 4 (Part (i), red color; Part (ii), green color) for a mixture of two Pareto distributions, as demonstrated in Example 3.

*respectively. As illustrated in Figure 2, the lower bound from Theorem 4, Part (i), offers a better approximation for this specific distribution compared to the lower bound from Part (ii).*

In many practical scenarios, the only available prior information may be that the component lifetimes exhibit a decreasing reversed failure rate (DRFR) property. A random variable X is said to satisfy the DRFR property if its reversed hazard rate function, τ(x), is decreasing in x>0.

**Theorem** **5.** *Let $X_{i},i=1,2,\ldots ,n$, be the i.i.d. lifetimes of components of a consecutive r-out-of-n:F systems with $T_{r|n:F}$ having the common RFR function τ(x). If X is DRFR, then*

$$-\frac{k}{2}E\left(\tau \left(T_{r|n:F}^{22}\right)\right)\leq J\left(T_{r|n:F}\right)\leq -\frac{2r-n}{2}E\left(\tau \left(T_{r|n:F}^{22}\right)\right),$$

*where* $T_{r|n:F}^{22}$ *has the pdf* $h_{r|n:F}^{22}(x)=2h_{r|n:F}(x)H_{r|n:F}(x)$, *for all* x>0.

**Proof.**  It is easy to see that the reversed hazard rate function of $T_{r|n:F}$ can be expressed as

$$\tau_{r|n:F}\left(x\right)=\psi_{r,n}\left(H\left(x\right)\right)\tau \left(x\right),$$

where

$$\psi_{r,n}(z)=\frac{r\left(n-r+1\right)-\left(r+1\right)\left(n-r\right)z}{\left(n-r+1\right)-\left(n-r\right)z},0<z<1.$$

Because $\psi_{r,n}^{\prime }(z)<0$ for 2r≥n and 0<z<1, it follows that $\psi_{r,n}(z)$ is a monotonically decreasing function of z. Given that $\psi_{r,n}(0)=r$ and $\psi_{r,n}(1)=2r-n$, we have 2r−n≤ $\psi_{r,n}(H(x))\leq k$ for 0<H(x)<1. This implies that $\left(2r-n)\tau (x)\leq \tau_{r|n:F}(x)\leq r\tau (x\right)$, for x>0. Combining this with (2) completes the proof. □

The following theorem applies under the assumption that the expectation of the squared reversed hazard rate function of X is finite.

**Theorem** **6.** *Under the conditions of Theorem 5, such that $E\left(\tau^{2}(X)\right)<\infty$, for 2r≥n, it holds that*

$$J\left(T_{r|n:F}\right)\geq -\frac{1}{2}\sqrt{\Omega_{r,n}E\left(\tau^{2}\left(X\right)\right)},\;\Omega_{r,n}=\int_{0}^{1}v^{2}g_{r|n:F}^{4}(v)dv.$$

**Proof.**  Note that the pdf of $T_{r|n:F}$ can be rewritten as $h_{r|n:F}(x)=h(x)g_{r|n:F}(H(x))$, its cumulative distribution function is $H_{r|n:F}(x)=G_{r|n:F}(H(x))$, and its RFR function is

$$\tau_{r|n:F}\left(x\right)=\tau \left(x\right)\frac{H\left(x\right)g_{r|n:F}\left(H\left(x\right)\right)}{G_{r|n:F}\left(H\left(x\right)\right)},\;for\;x>0.$$

Consequently, based on (2) and using Cauchy–Schwarz inequality, we obtain

$$\begin{array}{ll} \int_{0}^{\infty }\tau_{r|n:F}(x)h_{r|n:F}(x)H_{r|n:F}(x)dx & =\int_{0}^{\infty }\tau (x)\sqrt{h(x)}\sqrt{h(x)}H(x)g_{r|n:F}^{2}(H(x))dx\\ & \;\leq {\left(\int_{0}^{\infty }\tau^{2}(x)h(x)dx\right)}^{1/2}{\left(\int_{0}^{\infty }{\left(H(x)g_{r|n:F}^{2}(H(x))\right)}^{2}h(x)dx\right)}^{1/2}\\ & ={\left(E\left(\tau^{2}(X)\right)\right)}^{1/2}{\left(\int_{0}^{1}v^{2}g_{r|n:F}^{4}(v)dv\right)}^{1/2}. \end{array}$$

The last equality follows from the change of variable v=H(x), completing the proof. □

The following example demonstrates how the previously stated theorem can be applied to establish bounds for the extropy of a specific consecutive r-out-of-n:F system.

**Example** **4.** *Let us consider a system that fails if 6 consecutive components out of 8 components fail. Then, the lifetime of this system is $T_{6|8:F}$. We further assume that each component’s lifetime follows a Fréchet distribution, with the cdf given by*

$$H\left(x\right)=e^{-x^{-\alpha }},x>0,\;for\;\alpha >0.\;$$

*The reversed hazard rate function is given by* $\tau (x)=\alpha x^{-\alpha -1},x>0$, *and its second moment is* $E\left(\tau^{2}(X)\right)=\alpha^{2}\Gamma \left(\frac{1}{\alpha }+2\right)$. *Given that* $\Omega_{6,8}=21.1795$, *we obtain the following lower bound:*

$$J\left(T_{6|8:F}\right)\geq -2.3\alpha \sqrt{\Gamma \left(\frac{1}{\alpha }+2\right)}.$$

**Figure 3:**
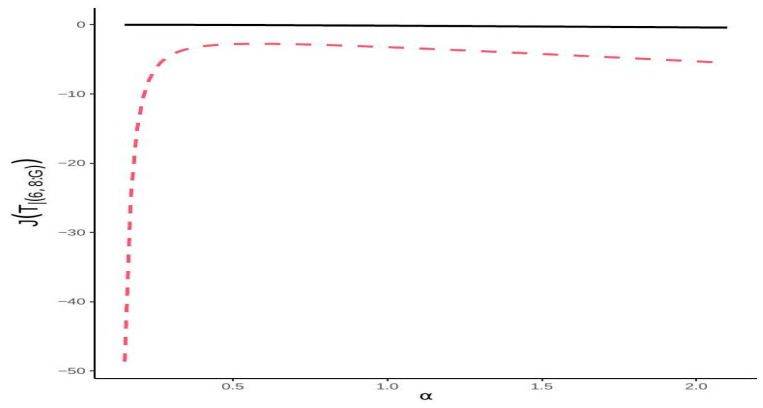
The exact values of $J\left(T_{6|8:F}\right)$ (solid line) and lower bounds (dashed line) from Theorem 6 for Fréchet distribution.

*Figure 3 illustrates the exact value and this lower bound.*

Unlike Theorem 6, we derived additional bounds for the extropy of consecutive *k*-out-of-*n*:F systems based on parallel system extropies. The proof is omitted as it follows directly from (7).

**Theorem** **7.** *For the consecutive r-out-of-n:F system with lifetime $T_{r|n:F}$, we have*

$$J\left(T_{r|n:F}\right)\geq (n-r+1{)}^{2}J\left(X_{r:r}\right)+(n-r{)}^{2}J\left(X_{r+1:r+1}\right),\;for\;2r\geq n.$$

## 4. Characterization Results

This section examines the extropy properties of consecutive r-out-of-*n*:F systems. Recent studies by Husseiny et al. [31] and Gupta and Chaudhary [32] have explored the characterization of symmetric continuous distributions using extropy and related measures, such as cumulative residual extropy and cumulative past extropy. Their findings indicate that for symmetric distributions, these measures yield identical values for both upper and lower order statistics. Building on these insights, we demonstrate that the extropy of the lifetime of a consecutive r-out-of-*n*:F system uniquely determines the underlying component distribution.

**Theorem** **8.** *Under the conditions of Theorem 2, $H_{X}$ and $H_{Y}$ belong to the same family of distributions but a change in location if and only if $X\leq_{\mathrm{disp}\;}Y$ and*

(10) $$J\left(T_{r|n:F}^{X}\right)=J\left(T_{r|n:F}^{Y}\right),\;\;\;\;\;for\;all\;r\;and\;n,\;such\;that\;2r\geq n,$$

*and* $g_{r:n|F}(v)>0$ *for all* 0<v<1.

**Proof.**  As the necessity condition is straightforward, we proceed to demonstrate the sufficiency condition. To prove sufficiency, we note that the extropy of $T_{r|n:F}^{X}$ from (6), can be expressed as follows:

$$J\left(T_{r|n:F}^{X}\right)=-\frac{1}{2}\int_{0}^{1}g_{r|n:F}^{2}(v)h_{X}\left(H_{X}^{-1}(v)\right)dv.$$

In a similar way, $J\left(T_{r|n:F}^{Y}\right)$ can be obtained. Assuming (10) holds for all 2r≥n, we have

(11) $$\int_{0}^{1}g_{r|n:F}^{2}(v)\left[h_{X}\left(H_{X}^{-1}(v)\right)-h_{Y}\left(H_{Y}^{-1}(v)\right)\right]dv=0.$$

For 2r≥n, it holds that $g_{r|n:F}^{2}(v)>0$ for all 0<v<1. Additionally, $X\leq_{\mathrm{disp}\;}Y$ implies $h_{X}\left(H_{X}^{-1}(v)\right)\geq h_{Y}\left(H_{Y}^{-1}(v)\right)$ for all 0<v<1 by (8). Thus, the integrand in (11) is nonnegative, implying

$$h_{X}\left(H_{X}^{-1}(v)\right)=h_{Y}\left(H_{Y}^{-1}(v)\right),\;a.e.\;v\in (0,1).$$

Thus, $H_{Y}^{-1}(v)=H_{X}^{-1}(v)+d$ for some constant d, which completes the proof. □

Since a consecutive n-out-of-n:F system functions as a parallel system, the following corollary follows directly from the preceding theorem.

**Corollary** **2.** *For two parallel systems with lifetimes $T_{n|n:F}^{X}$ and $T_{n|n:F}^{Y}$, then $H_{X}$ and $H_{Y}$ belong to the same family of distributions but a change in location if and only if $X\leq_{\mathrm{disp}\;}Y$ and*

$$J\left(T_{n|n:F}^{X}\right)=J\left(T_{n|n:F}^{Y}\right),\;\mathrm{for}\;\mathrm{all}\;n\geq 1.$$

The next theorem gives us another way to look at the same idea.

**Theorem** **9.** *Under the conditions of Theorem 8, $H_{X}$ and $H_{Y}$ belong to the same family of distributions but a change in location and scale if and only if $X\leq_{\mathrm{disp}\;}Y$ and*

(12) $$\frac{J\left(T_{r|n:F}^{X}\right)}{J(X)}=\frac{J\left(T_{r|n:F}^{Y}\right)}{J(Y)},\;for\;all\;r\;and\;n,\;such\;that\;2r\geq n.$$

**Proof.**  As the necessity condition is straightforward, we proceed to demonstrate the sufficiency condition. To prove sufficiency, from (6) we have

(13) $$\frac{J\left(T_{r|n:F}^{X}\right)}{J(X)}=-\frac{1}{2}\int_{0}^{1}g_{r|n:F}^{2}\left(v\right)\frac{h_{X}\left(H_{X}^{-1}\left(v\right)\right)}{J\left(X\right)}dv.$$

In a similar way, $J\left(T_{r|n:F}^{Y}\right)/J(Y)$ can be obtained. From (12) and (13), 2r≥n can be written as

(14) $$\int_{0}^{1}g_{r|n:F}^{2}\left(v\right)\frac{h_{X}\left(H_{X}^{-1}\left(v\right)\right)}{J\left(X\right)}dv=\int_{0}^{1}g_{r|n:F}^{2}\left(v\right)\frac{h_{Y}\left(H_{Y}^{-1}\left(v\right)\right)}{J\left(Y\right)}dv.$$

Let us set c=J(Y)/J(X), where J(X) and J(Y) denote the extropies of X and Y, respectively. Assumption $X\leq_{\mathrm{disp}\;}Y$ results in J(X)≤J(Y), which means that c≥1. Additionally, relation (14) can be written as

(15) $$\int_{0}^{1}g_{r|n:F}^{2}(v)\left[ch_{X}\left(H_{X}^{-1}\left(v\right)\right)-h_{Y}\left(H_{Y}^{-1}\left(v\right)\right)\right]dv=0.$$

Assumption $X\leq_{\mathrm{disp}\;}Y$ implies that $h_{X}\left(H_{X}^{-1}(v)\right)\geq h_{Y}\left(H_{Y}^{-1}(v)\right)$, or equivalently, $ch_{X}\left(H_{X}^{-1}(v)\right)\geq$ $h_{Y}\left(H_{Y}^{-1}(v)\right)$, for all 0<v<1, since c≥1. Hence, the integrand of (15) is strictly positive. Therefore, we have $H_{Y}^{-1}(v)=cH_{X}^{-1}(v)+d$ for some constant d, which completes the proof. □

As a direct consequence of Theorem 9, the following corollary holds.

**Corollary** **3.** *Under the conditions of Corollary 2, $H_{X}$ and $H_{Y}$ belong to the same family of distributions but a change in scale if and only if $X\leq_{\mathrm{disp}\;}Y$ and*

$$\frac{J\left(T_{n|n:F}^{X}\right)}{J(X)}=\frac{J\left(T_{n|n:F}^{Y}\right)}{J(Y)},\;\;for\;all\;n\geq 1.$$

We now present a novel characterization of consecutive systems using extropy. To this end, we examined a linear consecutive (n−i)-out-of-n:F system under the condition n≥2i, where i=0,1,…,n/2. As a foundational step, we revisited a lemma derived from the Müntz–Szász theorem, as referenced in Kamps [33], which plays a crucial role in moment-based characterization theorems.

**Lemma** **1.** *For an integrable function ψ(x) on the finite interval (a,b) if $\int_{a}^{b}x^{n_{j}}\psi (x)dx=$ 0,j≥1, then ψ(x)=0 for almost all x∈(a,b), where $\left\{n_{j},j\geq 1\right\}$ is a strictly increasing sequence of positive integers satisfying $\sum_{j=1}^{\infty }\frac{1}{n_{j}}=\infty$.*

*It is important to note that Lemma 1 is a well-established concept in functional analysis, stating that the sets* $\left\{x^{n_{1}},x^{n_{2}},\ldots ;1\leq n_{1}<n_{2}<\ldots \right\}$ *constitute a complete sequence. Notably, Hwang and Lin* [34] *expanded the scope of the Müntz–Szász theorem for the functions* $\left\{\phi^{n_{j}}(x),n_{j}\geq 1\right\}$*, where* ϕ(x) *is both absolutely continuous and monotonic over the interval* (a,b).

**Theorem** **10.** *Consider two consecutive (n−i )-out-of-n:F systems with lifetimes $T_{n-i|n:F}^{X}$ and $T_{n-i|n:F}^{Y}$ composed ofn i.i.d. components with cdfs $H_{X}$ and $H_{Y}$, and pdfs $h_{X}$ and $h_{Y}$, respectively. Then, $H_{X}$ and $H_{Y}$ belong to the same family of distributions but differ in location if and only if for a fixed i≥0,*

$$J\left(T_{n-i|n:F}^{X}\right)=J\left(T_{n-i|n:F}^{Y}\right),\;for\;all\;n\geq 2i.$$

**Proof.**  For the necessity part, since $H_{X}$ and $H_{Y}$ belong to the same family of distributions but differ in location, then $H_{Y}(y)=H_{X}(y-a)$ for all y≥a and a∈R. Then, it is clear that

$$\begin{array}{ll} J\left(T_{n-i|n:F}^{Y}\right) & =-\frac{1}{2}\int_{a}^{\infty }h_{Y,n-i|n:F}^{2}(y)dy=-\frac{1}{2}\int_{a}^{\infty }h_{X,n-i|n:F}^{2}(y-a)dy\\ & =-\frac{1}{2}\int_{0}^{\infty }h_{X,n-i|n:F}^{2}\left(x\right)dx=J\left(T_{n-i|n:F}^{X}\right).\left(\;\mathrm{taking}\;x=y-a\right). \end{array}$$

To establish the sufficiency part, we first note that for a consecutive (n−i)-out-of-n:F system, the following equation holds:

(16) $$g_{n-i|n:F}(v)=(n-i)(i+1)v^{n-i+1}-i(n-i+1)v^{n-i},0<v<1,$$

where n≥2i and i ranges from 1 to n/2. Given the assumption that $J\left(T_{n-i|n:F}^{X}\right)=J\left(T_{n-i|n:F}^{Y}\right)$, we can write

$$\int_{0}^{1}g_{n-i|n:F}^{2}(v)\left(h_{X}\left(H_{X}^{-1}\left(v\right)\right)-h_{Y}\left(H_{Y}^{-1}\left(v\right)\right)\right)dv=0,$$

or equivalently,

$$\int_{0}^{1}v^{n-2i}\phi_{i,n}(v)\left(h_{X}\left(H_{X}^{-1}\left(v\right)\right)-h_{Y}\left(H_{Y}^{-1}\left(v\right)\right)\right)dv=0,$$

where

$$\phi_{i,n}(v)=v^{n}[(n-i)(i+1)-i(n-i+1)v{]}^{2},\;for\;0<v<1.$$

By applying Lemma 1 with the function

$$\psi (v)=\phi_{i,n}(v)\left(h_{X}\left(H_{X}^{-1}(v)\right)-h_{Y}\left(H_{Y}^{-1}(v)\right)\right),$$

and considering the complete sequence $\left\{v^{n-2i},n\geq 2i\right\}$, one can conclude that

$$h_{X}\left(H_{X}^{-1}(v)\right)=h_{Y}\left(H_{Y}^{-1}(v),\;a.e.\;v\in (0,1).\right.$$

This implies that $H_{Y}^{-1}(x)=H_{X}^{-1}(x)+d$ for a constant d, completing the proof. □

In the special case where i=0, which corresponds to an n-out-of-n:F or parallel system, the following corollary holds.

**Corollary** **4.** *Under the conditions of Corollary 2, $H_{X}$ and $H_{Y}$ belong to the same family of distributions but differ in location if and only if*

$$J\left(T_{n|n:F}^{X}\right)=J\left(T_{n|n:F}^{Y}\right),\;for\;all\;n\geq 1.$$

Another helpful characterization is provided in the following theorem.

**Theorem** **11.** *Under the conditions of Theorem 1* *0, $H_{X}$ and $H_{Y}$ belong to the same family of distributions but differ in location and scale if and only if for a fixed i≥0,*

(17) $$\frac{J\left(T_{n-i|n:F}^{X}\right)}{J(X)}=\frac{J\left(T_{n-i|n:F}^{Y}\right)}{J(Y)},\;for\;all\;n\geq 2i.$$

**Proof.**  As the necessity is straightforward, we need to establish the sufficiency aspect. Leveraging Equations (1) and (17), we can derive

(18) $$\frac{J\left(T_{n-i|n:F}^{X}\right)}{J(X)}=-\frac{1}{2}\int_{0}^{1}g_{n-i|n:F}^{2}\left(v\right)\frac{h_{X}\left(H_{X}^{-1}\left(v\right)\right)}{J\left(X\right)}dv.$$

An analogous argument can be made for $J\left(T_{n-i|n:F}^{Y}\right)/J(X)$. If relation (17) holds for two cdfs $H_{X}$ and $H_{Y}$, then we can infer from Equation (18) that

$$\int_{0}^{1}g_{n-i|n:F}^{2}\left(v\right)\frac{h_{X}\left(H_{X}^{-1}\left(v\right)\right)}{J\left(X\right)}dv=\int_{0}^{1}g_{n-i|n:F}^{2}\left(v\right)\frac{h_{Y}\left(H_{Y}^{-1}\left(v\right)\right)}{J\left(Y\right)}dv.$$

Let us set

$$c=\frac{J(Y)}{J(X)}=\frac{\int_{0}^{1}h_{Y}\left(H_{Y}^{-1}(z)\right)dz}{\int_{0}^{1}h_{X}\left(H_{X}^{-1}(z)\right)dz}.$$

Using similar arguments as in the proof of Theorem 11, we can write

$$\int_{0}^{1}v^{n-2i}\phi_{i,n}v\left(ch_{X}\left(H_{X}^{-1}v\right)-h_{Y}\left(H_{Y}^{-1}v\right)\right)dv=0.$$

The proof is then completed by using similar arguments to those in Theorem 11. □

By applying Theorem 13, we derive the following corollary.

**Corollary** **5.** *Suppose the assumptions of Corollary 2. $H_{X}$ and $H_{Y}$ belong to the same family of distributions but differ in location and scale if and only if*

$$\frac{J\left(T_{n|n:F}^{X}\right)}{J(X)}=\frac{J\left(T_{n|n:F}^{Y}\right)}{J(Y)},\;for\;all\;n\geq 1.$$

The following theorem provides a characterization of the uniform distribution based on the extropy of consecutive (n−i)-out-of-n:F systems.

**Theorem** **12.** *Let us assume that $T_{n-i|n:F}$ is the lifetime of a consecutive (n−i)-out-of-n:F system, where the n i.i.d. component lifetimes have a pdf h, concentrated on (0,1). Then, X has a uniform distribution on (0,1) if and only if for a fixed i≥0,*

$$J\left(T_{n-i|n:F}^{X}\right)=-2J\left(X\right)J\left(U_{n-i|n:F}\right),\;for\;all\;n\geq 2i.$$

**Proof.**  Assuming X has a uniform distribution on (0,1), we have h(x)=1 for 0<x< 1, and therefore, for all $0<v<1,h\left(H^{-1}(v)\right)=1$. Consequently, Theorem 1 implies that $J\left(T_{n-i|n:F}^{X}\right)=J\left(U_{n-i|n:F}\right)$ for all n≥2i. Moreover, it is easy to see that J(X)=−1/2; therefore, the necessity is obtained. To prove the sufficiency part, let us assume that for all i, $J\left(T_{n-i|n:F}\right)=-2J(X)J\left(U_{n-i|n:F}\right)$, or equivalently,

(19) $$\int_{0}^{1}g_{n-i|n:F}^{2}(v)\left(h\left(H^{-1}\left(v\right)\right)+2J\left(X\right)\right)dv=0,$$

which holds when n≥2i, where $g_{n-i|n:F}(v)$ is defined in (16). Employing this, relation (19) can be rewritten as follows:

$$\int_{0}^{1}g_{n-i|n:F}^{2}(v)\left(h\left(H^{-1}(v)\right)+2J(X)\right)dv=0$$

or equivalently, we can obtain

$$\int_{0}^{1}v^{n-2i}\phi_{i,n}\left(v\right)\left(h\left(H^{-1}\left(v\right)\right)+2J\left(X\right)\right)dv=0,\;\mathrm{for}\;n\geq 2i.$$

By applying Lemma 1 with the function

$$\psi \left(v\right)=\phi_{i,n}\left(v\right)\left(h\left(H^{-1}\left(v\right)\right)+2J\left(X\right)\right),$$

and considering the complete sequence $\left\{v^{n-2i},n\geq 2i\right\}$, one can conclude that

$$h\left(H^{-1}(v)\right)=-2J(X),\;a.e.\;v\in (0,1).$$

This implies that

$$\frac{dH^{-1}}{dv}=\frac{1}{h\left(H^{-1}(v)\right)}=\frac{1}{-2J(X)}$$

Integrating both sides of the above equation from 0 to x, we find that $H^{-1}(x)=\frac{x}{-2J(X)}+d$ for some constant d, where x∈(0,1). Since $\underset{x\to 0}{lim}H^{-1}(x)=0$, it follows that d=0, leading to $H^{-1}(x)=\frac{x}{-2J(X)}$ for x∈(0,1). Given that $H^{-1}(1)=1$, we must have −2J(X)=1. Therefore, we conclude that H(x)=x for 0<x<1, indicating that X has a uniform distribution on (0,1), thus completing the proof. □

## 5. Nonparametric Estimation

In this section, we introduce a nonparametric estimation technique for the extropy of a consecutive (n−i)-out-of-n:F system. We consider i.i.d. absolutely continuous, non-negative random variables $X_{1},X_{2},\ldots ,X_{N}$, and their corresponding order statistics $X_{1:N}\leq X_{2:N}\leq \ldots \leq X_{N:N}$. Leveraging the identity $dH^{-1}(v)/dv=1/h\left(H^{-1}(v)\right),0<v<1$, from Equation (6), the extropy of $T_{n-i|n:F}$ can be formulated as follows:

(20) $$\begin{array}{l} J\left(T_{n-i|n:F}\right)=-\frac{1}{2}\int_{0}^{1}g_{n-i|n:F}^{2}\left(v\right)h\left(H^{-1}\left(v\right)\right)dv\\ =-\frac{1}{2}\int_{0}^{1}g_{n-i|n:F}^{2}(v)h\left(H^{-1}(v)\right)dv,\;for\;2r\geq n. \end{array}$$

To estimate the extropy $J\left(T_{n-i|n:F}\right)$ of the consecutive (n−i-out-of-n:F system, we utilize a difference operator-based estimator proposed by Vasicek [35] to approximate $\frac{dH^{-1}(v)}{dv}$. This estimator employs the empirical distribution function to estimate the distribution function of the random variable in (20). As a result, the following estimator for $J\left(T_{n-i|n:F}\right)$ is obtained:

(21) $$\begin{array}{l} \hat{J}\left(T_{n-i|n:F}\right)=-\frac{1}{2N}\sum_{i=1}^{N}{\left(g_{n-i|n:F}\left(\frac{i}{N+1}\right)\right)}^{2}\frac{2m}{N\left(X_{i+m:N}-X_{i-m:N}\right)}\\ \;\;=-\frac{1}{2N}\sum_{i=1}^{N}{\left(r\left(n-r+1\right){\left(\frac{i}{N+1}\right)}^{r-1}-\left(r+1\right)\left(n-r\right){\left(\frac{i}{N+1}\right)}^{r}\right)}^{2}\\ \;\;\;\;\;\;\times \frac{2m}{N\left(X_{i+m:N}-X_{i-m:N}\right)} \end{array}$$

where m represents a positive integer less than N/2, defined as the window size. For cases where $i-m\leq 1,X_{i-m:N}$ is set to $X_{1:N}$, and for $i+m\geq N,X_{i+m:N}$ is set to $X_{N:N}$. To see how well our estimator works, we first use it as a simple example. We assume that the component lifetimes follow an exponential distribution of a pdf given by $h(x)=e^{-x}$ for x>0. Under this assumption, $J\left(T_{n-i|n:F}\right)$ can be computed as follows:

$$\begin{array}{ll} J\left(T_{n-i|n:F}\right) & =-\frac{1}{2}\int_{0}^{1}g_{n-i|n:F}^{2}(v)(1-v)dv\\ & =-\frac{1}{4}\left[\frac{\left(n-i\right)\left(n-3i-1\right)+n\left(n+2\right)}{\left(2\left(n-i\right)-1\right)\left(2\left(n-i\right)+1\right)}\right],\;for\;n\geq 2i. \end{array}$$

The second case involves a uniform distribution with a pdf defined as h(x)=1 for 0<x<1. For this distribution, $J\left(T_{n-i|n:F}\right)$ can be determined as follows:

$$\begin{array}{ll} J\left(T_{n-i|n:F}\right) & =J\left(U_{n-i|n:F}\right)=-\frac{1}{2}\int_{0}^{1}g_{n-i|n:F}^{2}(v)dv\\ & =-\frac{1}{2}\left[\frac{((n-i)(i+1){)}^{2}}{2\left(n-i\right)-1}+\frac{((n-i+1)i{)}^{2}}{2\left(n-i\right)+1}-i\left(i+1\right)\left(n-i+1\right)\right],\;for\;n\geq 2i. \end{array}$$

The average bias and root mean square error (RMSE) of the estimators for these distributions are evaluated using Equation (21). To this end, we computed the bias and RMSE for various sample sizes, N = 20, 30, 40, 50, 100 and different values of i and n. For simplicity, we used the heuristic formula from [36] to determine the parameter m, given by

$$m=[\sqrt{N}+0.5]$$

Based on 5000 repetitions, the results are summarized in Table 1 and Table 2. As the sample size increased, the RMSE of the extropy estimators for the consecutive (n−i)-out-of-n:F system decreased, while the bias increased. This observation suggests that larger sample sizes yielded more precise estimates, albeit with a slight increase in bias.

### 5.1. Test of Uniformity

Testing for uniformity is a fundamental statistical task with applications across various fields. Consider a random sample $X_{1},X_{2},\ldots ,X_{N}$ drawn from an absolutely continuous distribution H, with order statistics satisfying $X_{1:N}\leq X_{2:N}\leq \cdots \leq X_{N:N}$. Suppose that $H_{0}$ denotes the cumulative distribution function (cdf) of the standard uniform distribution $U\left(0,1\right)$, given by $H_{0}\left(x\right)=x$, for 0≤x≤1.

Our objective was to test the following hypothesis:

$$H_{0}:H\left(x\right)=H_{0}\left(x\right),\;\mathrm{vs}.\;H_{1}:H\left(x\right)\neq H_{0}\left(x\right).$$

As established in Theorem 14, the uniform distribution is uniquely characterized by the extropy of consecutive (n−i)-from-n: F systems. Building on this result, we introduced a new test statistic for uniformity, denoted by $TC_{i,n}$, which is defined as follows:

$$TC_{i,n}=J\left(T_{n-i|n:F}^{X}\right)+2J\left(X\right)J\left(U_{n-i|n:F}\right),\;for\;i=0,1,\ldots ,\lceil n/2\rceil .$$

Therefore, Theorem 13 directly implies that $TC_{i,n}=0$ if and only if X is uniformly distributed. Hence, $TC_{i,n}$ can serve as a basic measure of uniformity and be used as a test statistic. Given an estimator ${\hat{TC}}_{i,n}$ of $TC_{i,n}$ based on a random sample $X_{1},X_{2},\ldots ,X_{N}$, significant deviations of ${\hat{TC}}_{i,n}$ from its expected value under the null hypothesis suggest nonuniformity, leading to the rejection of $H_{0}$. For illustration purposes, we focus on the special case of n=4 and i=2, using ${\hat{TC}}_{2,4}$ as the test statistic. To derive an expression for ${\hat{TC}}_{2,4}$, we recall that Qiu and Jia’s [21] estimator for J(X), denoted as $JQ2_{mn}$, is defined by

$$JQ2_{mn}=-\frac{1}{2N}\sum_{l=1}^{N}\frac{c_{l}m}{N\left(X_{l+m:N}-X_{l-m:N}\right)},$$

where $c_{l}$ depends on the window size m and the sample size N and is defined as

(22) $$c_{l}=\left\{\begin{array}{ll} 1+\frac{l-1}{m} & \;\mathrm{if}\;1\leq l\leq m\\ 2 & \;\mathrm{if}\;m+1\leq l\leq N-m\\ 1+\frac{N-l}{m} & \;\mathrm{if}\;N-m+1\leq l\leq N \end{array}\right.$$

As $J\left(U_{2|4:F}\right)=-0.6$, a reasonable estimator for ${\hat{TC}}_{2,4}$ can be derived using Equation (21) and the $JQ2_{mn}$ estimator, as follows:

(23) $$\begin{array}{ll} {\hat{TC}}_{4,2} & =\hat{J}\left(T_{2|4:F}^{X}\right)+2JQ2_{mn}J\left(U_{2|4:F}\right)\\ & =-\frac{1}{2N}\sum_{l=1}^{N}{\left(g_{2|4:F}\left(\frac{l}{N+1}\right)\right)}^{2}\frac{2m}{N\left(X_{l+m:N}-X_{l-m:N}\right)}+\frac{1.2}{2N}\sum_{l=1}^{N}\frac{c_{l}m}{N\left(X_{l+m:N}-X_{l-m:N}\right)}\\ & =-\frac{1}{2N}\sum_{l=1}^{N}\left[72{\left(\left(\frac{l}{N+1}\right)-{\left(\frac{l}{N+1}\right)}^{2}\right)}^{2}-1.2c_{l}\right]\sum_{l=1}^{N}\frac{c_{l}m}{N\left(X_{l+m:N}-X_{l-m:N}\right)}. \end{array}$$

Ensuring the consistency of an estimator is essential, particularly when evaluating estimators for parametric functions. The following theorem establishes the results presented in Equation (23). Its proof follows a methodology similar to that of Theorem 1 in Vasicek’s paper [35]. Notably, Park [37] and Xiong et al. [38] adopted Vasicek’s approach to demonstrate the consistency of their respective test statistics.

**Theorem** **13.** *Assume that $X_{1},X_{2},\ldots ,X_{N}$ is a random sample of size N taken from a population with pdf h and cdf H. Also, let the variance of the random variable be finite. Then, ${\hat{TC}}_{4,2}\overset{p\;}{\to }TC_{4,2}$ as N→+∞,m→+∞ and $\frac{m}{N}\to 0$, where $\overset{p\;}{\to }$ stands for the convergence in probability.*

**Proof.**  Part (2) of Theorem 2.1 in Qiu and Jia [21] establishes that $JQ2_{mn}\overset{p}{\to }J(X)$ as N→+∞,m→+∞ and $\frac{m}{N}\to 0$. Furthermore, by adapting the approach of Theorem 1 in Vasicek [35], it can be shown that $\hat{J}\left(T_{2|4:F}^{X}\right)\overset{p}{\to }J\left(T_{2|4:F}^{X}\right)$ under the same asymptotic conditions. Consequently, leveraging the properties of convergence in probability, we obtain ${\hat{TC}}_{4,2}\overset{p}{\to }TC_{4,2}$ as N→+∞,m→+∞ and $\frac{m}{N}\to 0$, completing the proof. □

The following theorem demonstrates that shifting the random variable X does not affect the RMSE of ${\hat{TC}}_{4,2}$ when estimating $TC_{4,2}$. However, this invariance does not extend to scale transformations. The proof of these results follows directly from the arguments presented by Ebrahimi et al. [39].

**Theorem** **14.** *Assume that $X_{1},X_{2},\ldots ,X_{N}$ is a random sample of size N taken from a population with pdf h and cdfH and $Y_{j}=aX_{j}+b,a>0,b\in R$. Denote the estimators for $TC_{4,2}$ on the basis of $X_{j}$ and $Y_{j}$ with ${\hat{TC}}_{4,2}^{X}$ and ${\hat{TC}}_{4,2}^{Y}$, respectively. Then, the following properties apply:*

(i) $E\left({\hat{TC}}_{4,2}^{Y}\right)=E\left({\hat{TC}}_{4,2}^{X}\right)/a$,
(ii) $Var\left({\hat{TC}}_{4,2}^{Y}\right)=Var\left({\hat{TC}}_{4,2}^{X}\right)/a^{2}$,
(iii) $RMSE\left({\hat{TC}}_{4,2}^{Y}\right)=RMSE\left({\hat{TC}}_{4,2}^{X}\right)/a$.

**Proof.**  It is not hard to see from (23) that

$$\begin{array}{ll} {\hat{TC}}_{4,2}^{Y} & =-\frac{1}{2N}\sum_{l=1}^{N}\left[72{\left(\left(\frac{l}{N+1}\right)-{\left(\frac{l}{N+1}\right)}^{2}\right)}^{2}-1.2c_{l}\right]\frac{m}{N\left(Y_{l+m:N}-Y_{l-m:N}\right)}\\ & =-\frac{1}{2N}\sum_{l=1}^{N}\left[72{\left(\left(\frac{l}{N+1}\right)-{\left(\frac{l}{N+1}\right)}^{2}\right)}^{2}-1.2c_{l}\right]\frac{m}{Na\left(X_{l+m:N}-X_{l-m:N}\right)}\\ & =\frac{{\hat{TC}}_{4,2}^{X}}{a} \end{array}$$

The proof is then completed by leveraging the properties of the mean, variance, and RMSE of ${\hat{TC}}_{4,2}^{Y}={\hat{TC}}_{4,2}^{X}/a$. □

The absolute value of ${\hat{TC}}_{4,2}$ converges to zero as the sample size N approaches infinity under the null hypothesis $H_{0}$. Conversely, under an alternative distribution on [0,1], with an absolutely continuous cdf H, the absolute value of ${\hat{TC}}_{4,2}$ reaches a value greater than zero with probability sa N→+∞. Based on these properties, we reject the null hypothesis for any significance level α, and a finite sample size N, if the test statistic $\left|{\hat{TC}}_{4,2}\right|$ exceeds the critical value ${\left|{\hat{TC}}_{4,2}\right|}_{\left(1-\alpha \right)}$. Since the values of $\left|{\hat{TC}}_{4,2}\right|$ are influenced by the sample size and the window parameter m, its asymptotic distribution is complex and not easily analyzable theoretically. To address this, we employed the Monte Carlo method, generating 10,000 samples of sizes N=5,10,20,30,40,50,100 from the null distribution and determined the (1−α)-th quantile to serve as the critical value for the significance level α. We selected m using the heuristic formula $m=\left[\frac{N}{2}\right]-1.$

Table 3 and Table 4 present the critical values corresponding to different sample sizes at significance levels α=0.1,0.05,0.01.

### 5.2. Power Comparisons

**Figure 4:**
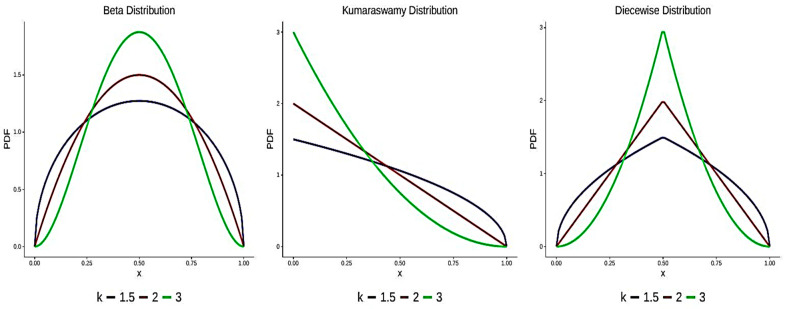
The probability density functions of $A_{k},B_{k},$ and $C_{k}$ distributions.

To calculate the power of the tests, a random sample assuming all possible values in the interval (0,1) was generated from non-standard uniform distributions, such as Beta, Kumaraswamy, and piecewise distributions (see Cordeiro and De Castro [40], whose supports varied between 0 and 1). The Monte Carlo study of the proposed test $\left|{\hat{TC}}_{4,2}\right|$ was performed under nine alternative distributions. For each sample size N,10,000 samples of size N were generated from the alternative distributions, and the statistic $\left|{\hat{TC}}_{4,2}\right|$ was then calculated. For the level α, the power of $\left|{\hat{TC}}_{4,2}\right|$ was estimated by the proportion of 10,000 samples that fell within the critical range. The distribution functions of the alternatives considered were as follows:

- $A_{k}:H(x)=I_{x}(k,k),0\leq x\leq 1$,
- $B_{k}:H(x)=1-(1-x{)}^{k},0\leq x\leq 1$,
- $C_{k}:H(x)=\left\{\begin{array}{ll} 2^{k-1}x^{k} & \;\mathrm{if}\;0\leq x\leq 0.5\\ 1-2^{k-1}(1-x{)}^{k} & \;\mathrm{if}\;0.5<x\leq 1 \end{array}\right.,$
  for k=1.5,2,3, where
  $$I_{x}(a,b)=\frac{B(x;a,b)}{B(a,b)},B(x;a,b)=\int_{0}^{x}t^{a-1}(1-t{)}^{b-1}dt$$

denotes the regularized incomplete beta function, and B(a,b) is the complete beta function. Figure 4 illustrates the pdfs of the alternative hypotheses $A_{k},B_{k}$, and $C_{k}$. As evident from the figure, alternatives A and C are both more likely to produce values closer to 0.5 than the uniform distribution. However, alternative C exhibits a stronger concentration around 0.5, suggesting a higher probability of observing values very near the midpoint. Alternative A, on the other hand, tends to produce values closer to 0 than expected under the null hypothesis. To evaluate the performance of our proposed test statistic, we compare its power to that of several widely used statistics under the same alternative hypotheses. These include the following:

- The Kolmogrov–Smirnov statistic (Kolmogorov [41] and Smirnov [42]):
  $$KS=max\left(\underset{1\leq l\leq N}{max}\left(\frac{l}{N}-X_{l:N}\right),\underset{1\leq l\leq N}{max}\left(X_{l:N}-\frac{l-1}{N}\right)\right),$$
- The Anderson–Darling statistic (Anderson and Darling [43]):
  $$AD=-\sum_{l=1}^{N}\frac{2l-1}{N}\left[logX_{l:N}+log\left(1-X_{N-l+1:N}\right)\right]-N,$$
- The Cramér–von Mises statistic (Cramér [44]; von Mises [45]):
  $$CM=\sum_{l=1}^{N}{\left(\frac{2l-1}{2N}-X_{l:N}\right)}^{2}+\frac{1}{12N},$$
- The Dudewicz and Van der statistic:
  $$ENT=\frac{1}{N}\sum_{l=1}^{N}{log}_{2}\frac{2m}{N\left(X_{l+m:N}-X_{l-m:N}\right)},$$
- The Kuiper statistic:
  $$V=\underset{1\leq l\leq N}{max}\left(\frac{l}{N}-X_{l:N}\right)+\underset{1\leq l\leq N}{max}\left(X_{l:N}-\frac{l-1}{N}\right),$$
- The Qiu and Jia statistic (Qiua and Jia [21]):
  $$TU=-JQ2_{mn}=\frac{1}{2N}\sum_{l=1}^{N}\frac{c_{l}m}{N\left(X_{l+m:N}-X_{l-m:N}\right)},$$

where $c_{l}$ is defined in (22).

**Figure 5:**
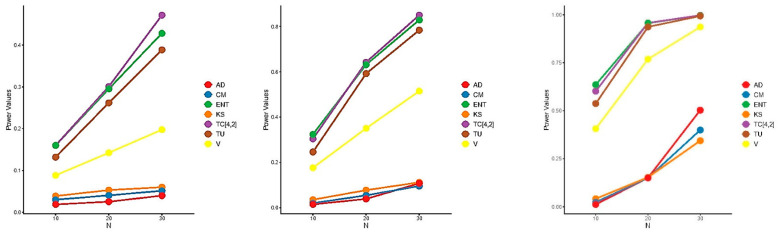
Power comparisons of the test statistics for $A_{1.5}$ (**left**), $A_{2}$ (**middle**), and $A_{3}$ (**right**) at significance level α=0.05.

**Figure 6:**
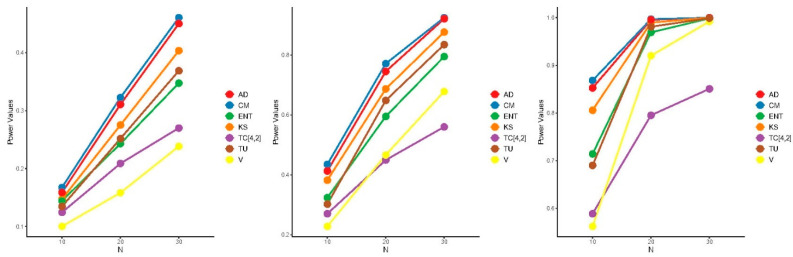
Power comparisons of the test statistics for $B_{1.5}$ (**left**), $B_{2}$ (**middle**), and $B_{3}$ (**right**) at significance level α=0.05.

**Figure 7:**
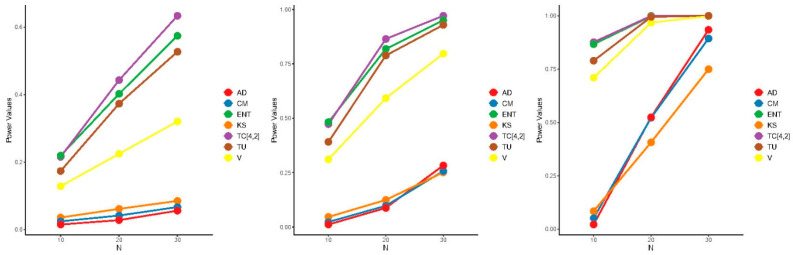
Power comparisons of the test statistics for $C_{1.5}$ (**left**), $C_{2}$ (**middle**), and $C_{3}$ (**right**) at significance level α=0.05.

The power of the proposed test is influenced by both the window size m and the specific alternative distribution, making it challenging to determine the optimal value of m that maximizes power across all alternatives. Consequently, we adopted the heuristic formula $m=\left[\frac{N}{2}\right]-1$, to select m, aiming for good (though not necessarily optimal) power across all alternative distributions. Figure 5, Figure 6 and Figure 7 present the results of the power comparisons. As shown in these figures, our ${\hat{TC}}_{4,2}$ test exhibited strong performance against alternative A, especially as the sample size N increased. However, it performed less favorably against alternative B. For alternative C, our ${\hat{TC}}_{4,2}$ test also performed well, with comparable performance to the Dudewicz and Van der Meulen ENT test for $C_{3}$.

**Example** **5.** **(Real data application).** *As discussed in Illowsky and Dean [46] on page 317, Table 5.1, we consider a dataset containing the smiling times, in seconds, of 55 babies. These smiling times are assumed to follow a uniform distribution between 0 and 23 s, inclusive.*

**Dataset:** 10.4, 19.6, 18.8, 13.9, 17.8, 16.8, 21.6, 17.9, 12.5, 11.1, 4.9, 12.8, 14.8, 22.8, 20.0, 15.9, 16.3, 13.4, 17.1, 14.5, 19.0, 22.8, 1.3, 0.7, 8.9, 11.9, 10.9, 7.3, 5.9, 3.7, 17.9, 19.2, 9.8, 5.8, 6.9, 2.6, 5.8, 21.7, 11.8, 3.4, 2.1, 4.5, 6.3, 10.7, 8.9, 9.4, 9.4, 7.6, 10.0, 3.3, 6.7, 7.8, 11.6, 13.8, 18.6.

This implies that any smiling time between 0 and 23 s, inclusive, is equally likely. By applying the transformation $x_{i}/23$, to the given data $x_{l},l=1,2,\ldots ,55$, we standardized the data to a uniform distribution on the interval (0,1). For these transformed data, the calculated value of the test statistic was $\left|{\hat{TC}}_{4,2}\right|=0.1522$, while the critical value at the 0.05 significance level was ${\left|{\hat{TC}}_{4,2}\right|}_{0.95}=0.1582$. Since the test statistic fell within the acceptance region, we failed to reject the null hypothesis and conclude that the data follow a uniform distribution on (0,1).

## 6. Conclusions

Extropy and its various generalizations have emerged as powerful tools with widespread applications across diverse scientific and engineering fields, including information theory, economics, communication theory, and physics. For instance, negative cumulative extropy has been utilized in the analysis of stock markets in OECD countries by Tahmasebi and Toomaj [47], while Tsallis extropy has found application in pattern recognition by Balakrishnan et al. [48]. Furthermore, fractional Deng extropy has been applied to classification problems by Kazemi et al. [49], and various extropy measures have been employed in compressive sensing as shown by Tahmasebi et al. [50]. The objective of this study was to extend the understanding and application of extropy to the context of consecutive r-out-of-n:F systems. These systems are fundamental in reliability engineering and represent a critical class of systems, where failure occurs if r consecutive components fail. Understanding the information content and uncertainty within such systems, as quantified by extropy, is crucial for assessing their performance, reliability, and overall robustness. Our investigation successfully established a significant relationship between the extropy of consecutive r-out-of-n:F systems derived from continuous distributions and those obtained from uniform distributions, providing a foundational insight into their behavior. Recognizing the inherent challenges in deriving closed-form expressions for extropy, especially in scenarios involving a large number of system components or complex component distributions, we introduced a comprehensive range of useful bounds. These bounds offer practical tools for effectively estimating the extropy of consecutive r-out-of-n:F systems, even when exact calculations are intractable. Furthermore, we proposed a novel extropy estimator specifically tailored for consecutive r-out-of-n:F systems, designed for direct application in practical settings. As a compelling example of its practical utility, we showcased the application of the proposed extropy estimator for a goodness-of-fit test for the standard uniform distribution. While many existing test statistics for assessing uniformity are built upon other certainty measures, such as those discussed by Blinov and Lemeshko [51], Mohamed et al. [52,53] using fractional entropy and cumulative residual Tsallis entropy, and Noughabi [54] applying cumulative residual entropy, the use of extropy offers distinct advantages. In the context of consecutive systems, an extropy-based test can be particularly sensitive to deviations from uniformity that might manifest as specific patterns, which are inherently captured by number of components and *r* consecutive failed components as a parameter of flexibility. This provides a complementary and potentially more nuanced perspective on assessing uniformity, especially when the underlying data might exhibit characteristics relevant to system reliability or sequence-dependent events. The extropy-based test provides an alternative and robust method for evaluating the fit of data to a uniform distribution, leveraging the unique properties of extropy in capturing information content.

## Figures and Tables

**Table 1:** The bias and RMSE values of the estimate of $J\left(T_{n-i|n:F}\right),$ with standard exponential distribution component lifetimes for different choices of i and n.

*N* = 20				*N* = 30		*N* = 40		*N* = 50		*N* = 100	
*n*	*i*	Bias	RMSE	Bias	RMSE	Bias	RMSE	Bias	RMSE	Bias	RMSE
5	0	−0.145895	0.050749	−0.089774	0.018277	−0.059770	0.009551	−0.044811	0.005837	−0.011864	0.001097
	1	−0.078625	0.018531	−0.041885	0.006271	−0.026529	0.003121	−0.017465	0.001909	−0.003345	0.000571
	2	−0.036039	0.006134	−0.018140	0.003219	−0.012438	0.002164	−0.009271	0.001516	−0.004116	0.000757
6	0	−0.177926	0.071332	−0.111349	0.028405	−0.083625	0.015152	−0.064254	0.009374	−0.020052	0.001779
	1	−0.118696	0.031228	−0.066450	0.011488	−0.041432	0.005272	−0.029434	0.003241	−0.006391	0.000766
	2	−0.055588	0.011645	−0.026799	0.003965	−0.015054	0.002124	−0.009608	0.001451	−0.000843	0.000593
	3	−0.028573	0.007610	−0.019522	0.004667	−0.014865	0.003251	−0.009383	0.002604	−0.007302	0.001241
7	0	−0.215926	0.105451	−0.143378	0.041398	−0.105595	0.022722	−0.083527	0.015081	−0.027646	0.002861
	1	−0.151213	0.053749	−0.089206	0.020752	−0.061046	0.010124	−0.044897	0.006181	−0.011651	0.001151
	2	−0.087616	0.021155	−0.045373	0.007131	−0.026959	0.003476	−0.017064	0.002072	−0.001945	0.000608
	3	−0.037821	0.008069	−0.016963	0.003372	−0.008987	0.002098	−0.003187	0.001562	−0.000514	0.000770
8	0	−0.245875	0.133512	−0.169920	0.058762	−0.127069	0.031831	−0.101061	0.021011	−0.038070	0.004022
	1	−0.188737	0.081671	−0.116732	0.032998	−0.084663	0.016489	−0.063760	0.010117	−0.018165	0.001815
	2	−0.124965	0.036731	−0.068022	0.013715	−0.042473	0.006675	−0.031082	0.003656	−0.004798	0.000838
	3	−0.065324	0.015026	−0.028932	0.005053	−0.014906	0.002663	−0.007793	0.001549	0.001065	0.000666
	4	−0.024499	0.008091	−0.011365	0.003940	−0.005889	0.002853	−0.001748	0.002046	−0.002914	0.001145

**Table 2:** The bias and RMSE values of the estimate of $J\left(T_{n-i|n:F}\right),$ with standard uniform distribution component lifetimes for different choices of i and n.

*N* = 20				*N* = 30		*N* = 40		*N* = 50		*N* = 100	
*n*	*i*	Bias	RMSE	Bias	RMSE	Bias	RMSE	Bias	RMSE	Bias	RMSE
5	0	−0.936094	1.444092	−0.793851	1.144772	−0.713842	1.004388	−0.664756	0.905735	−0.512761	0.628195
	1	−0.590450	0.871130	−0.454252	0.658060	−0.386377	0.536204	−0.344834	0.463333	−0.238968	0.311527
	2	−0.260561	0.347903	−0.188247	0.236120	−0.147586	0.187416	−0.124121	0.159152	−0.075071	0.097070
6	0	−1.115787	1.785087	−0.966685	1.404569	−0.898500	1.260244	−0.849516	1.141714	−0.655093	0.859432
	1	−0.789329	1.243988	−0.640423	0.918773	−0.574205	0.796915	−0.527410	0.702302	−0.369448	0.471387
	2	−0.461131	0.652054	−0.335695	0.465028	−0.276477	0.379773	−0.235081	0.320505	−0.152461	0.199439
	3	−0.195613	0.259936	−0.131979	0.179150	−0.101657	0.142935	−0.082456	0.119049	−0.048043	0.078041
7	0	−1.300057	2.517281	−1.340618	2.220741	−1.390172	2.041344	−1.351307	1.832077	−1.174914	1.460273
	1	−0.974527	1.521404	−0.835748	1.250773	−0.752920	1.064358	−0.703541	0.932074	−0.515872	0.670624
	2	−0.663265	0.980888	−0.519456	0.705243	−0.435916	0.605943	−0.388588	0.509926	−0.257060	0.332286
	3	−0.347348	0.489964	−0.246278	0.340897	−0.191480	0.257628	−0.155908	0.216469	−0.094337	0.130185
8	0	−1.280009	2.387094	−1.292371	1.970096	−1.220243	1.730366	−1.173613	1.614975	−0.998048	1.256077
	1	−1.149068	1.823176	−1.009816	1.512126	−0.934285	1.290326	−0.866499	1.153410	−0.657747	0.844607
	2	−0.864234	1.323735	−0.723328	0.999044	−0.611858	0.838130	−0.560017	0.738741	−0.393868	0.510655
	3	−0.583059	0.817869	−0.411258	0.576096	−0.329835	0.453799	−0.285657	0.389149	−0.176501	0.234242
	4	−0.283538	0.391618	−0.192602	0.260096	−0.139642	0.198825	−0.111210	0.170093	−0.061858	0.107112

**Table 3:** Critical values of the $\left|{\hat{TC}}_{4,2}\right|$ statistic at significance level α=0.05.

m	N = 5	N = 10	N = 20	N = 30	N = 40	N = 50	N = 100
2	1.055246	0.378625	0.334115	0.292918	0.235903	0.214288	0.145114
3		0.291554	0.230632	0.203458	0.181164	0.164139	0.111786
4		0.333465	0.182454	0.170887	0.158504	0.142908	0.100424
5			0.162901	0.151537	0.138488	0.128358	0.092997
6			0.161813	0.136521	0.127321	0.119330	0.091328
7			0.165962	0.127548	0.117327	0.114573	0.086915
8			0.183542	0.122604	0.110561	0.106489	0.084083
9			0.207359	0.123269	0.105909	0.098579	0.081979
10				0.127054	0.105783	0.098879	0.081248
11				0.136003	0.103538	0.094049	0.077391
12				0.146328	0.105827	0.093091	0.076731
13				0.159688	0.108399	0.093009	0.074217
14				0.177465	0.113563	0.094735	0.073368
15					0.121417	0.095208	0.071351
16					0.128991	0.097564	0.069159
17					0.138696	0.102574	0.069402
18					0.150457	0.106580	0.068427
19					0.163477	0.112738	0.067897
20						0.119261	0.066284
21						0.126934	0.065713
22						0.135583	0.065328
23						0.145161	0.065742
24						0.155907	0.067071
25							0.066916
26							0.068518
27							0.068396
28							0.069368
29							0.070967
30							0.072728

**Table 4:** Critical values of the $\left|{\hat{TC}}_{4,2}\right|$ statistic at significance level α=0.01.

m	N = 5	N = 10	N = 20	N = 30	N = 40	N = 50	N = 100
2	1.743562	0.677543	0.658744	0.48241	0.434795	0.368653	0.229218
3		0.386052	0.353718	0.328039	0.282377	0.24862	0.158054
4		0.405918	0.25605	0.255527	0.227769	0.203122	0.139741
5			0.217423	0.208839	0.192097	0.180964	0.135573
6			0.194913	0.179679	0.178653	0.166450	0.125934
7			0.197127	0.167431	0.162625	0.155882	0.121372
8			0.207123	0.156101	0.153641	0.145689	0.114386
9			0.229525	0.151309	0.140698	0.130960	0.110536
10				0.148928	0.132649	0.128669	0.110754
11				0.152661	0.127555	0.124188	0.106882
12				0.161572	0.124451	0.117477	0.102301
13				0.175248	0.127549	0.116759	0.097866
14				0.189690	0.131132	0.113071	0.096778
15					0.135145	0.114522	0.094484
16					0.141079	0.113447	0.094325
17					0.149820	0.115286	0.092486
18					0.160194	0.119796	0.090843
19					0.173629	0.124119	0.089592
20						0.128747	0.087058
21						0.135821	0.085291
22						0.144409	0.084226
23						0.153875	0.083061
24						0.163402	0.083231
25							0.083082
26							0.083062
27							0.083960
28							0.084174
29							0.085061
30							0.084448

## Data Availability

All data generated or analyzed during this study are included in this published article.
